# F-18 FDG PET/CT Imaging of Eccrine Sweat Gland Carcinoma of the Scrotum with Extensive Regional and Distant Metastases

**DOI:** 10.22038/aojnmb.2017.19550.1145

**Published:** 2017

**Authors:** Jin-Suk Kim

**Affiliations:** Department of Nuclear Medicine, Konyang University Hospital, Daejeon, Republic of Korea

**Keywords:** Distant metastasis, Eccrine sweat gland carcinoma, F-18 FDG, PET/CT, Scrotum

## Abstract

Eccrine carcinoma is an extremely rare malignant skin cancer arising from eccrine sweat glands with a high metastatic potential. It mainly occurs in the elderly, with equal incidence in both sexes. It usually spreads to regional lymph nodes, with liver, lungs, and bones being the most common sites of distant metastasis. Because of tumor rarity, little is known about the value of F-18 FDG PET/CT in evaluating this disease. Our case report aims to increase current knowledge of F-18 FDG PET/CT in eccrine sweat gland carcinoma as a noninvasive imaging tool for assessing the extension of the disease and detecting distant metastases.

We reported a 96-year-old man who presented with as lowly progressive, ill-margined erythematous papules and nodules with a crusted and eroded involving multiple sites of groin, scrotum, penis, left pelvic wall, left hip and left thigh for >3 years, which became extensive in the past 2 months. The histologic investigation confirmed the diagnosis of an eccrine carcinoma. He was performed F-18 FDG PET/CT to further evaluate the lesions. FDG PET/CT imaging revealed FDG uptake at the extensive skin lesion, involvement of lymph nodes, and multiple FDG-avid of liver, skeletal and lung metastases.

## Introduction

Sweat gland carcinomas are rare malignant tumors that were first described in 1865 by Cornil (1, 2). Recent studies have classified sweat gland carcinomas into eccrine and apocrine tumors (3, 4), and these groups demonstrate the potential for local tissue infiltration, as well as regional and distant metastases. Because regional lymph node involvement and distant metastasis are indicators of poor prognosis, identification of these factors is crucial for successful management of the disease. Although F18-fluorodeoxyglucose positron emission tomography/computed tomography (F-18 FDG PET/CT) imaging is useful for nodal staging and distant metastasis detection in malignant tumors, its use in evaluating eccrine carcinoma is infrequently reported in the literature.

In this report, I describe the case of an elderly man with eccrine carcinoma of the scrotum with extensive regional and distant metastases, for whom F-18 FDG PET/CT was valuable in detecting distant metastasis and assessing the extent of the disease.

## Case report

A 96-year-old man with a 5-year history of end-stage renal disease and hypertension was admitted to Division of Nephrology with a complaint of left leg edema. Lower extremity ultrasonography showed no definitive evidence of deep vein thrombosis. Physical examination revealed extensive ill-margined erythematous papules and nodules with crusted and eroded surfaces involving multiple sites on the groin, scrotum, penis, left pelvic wall, left hip, and left thigh. Large inguinal lymph nodes were palpated.

**Figure 1:**
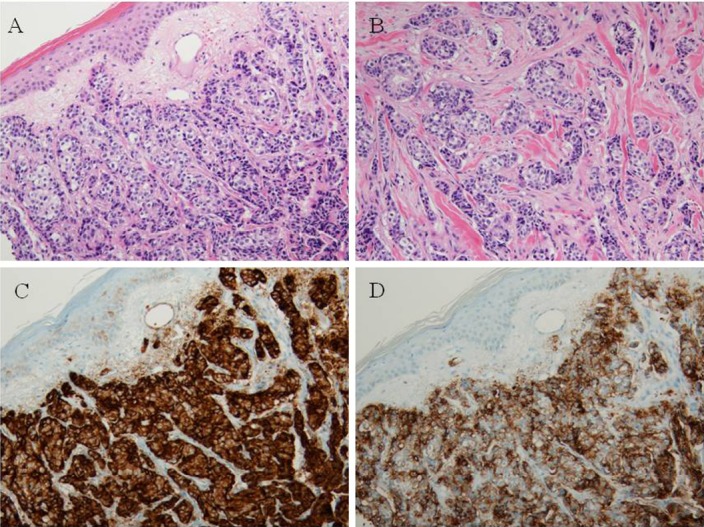
Histologic findings of the genital tumor. The tumor consists of solid nests and tubules extending from dermis (A and B, ×200). Immunohistochemistry reveals positive reactions for epithelial membrane antigen (C, ×200) and carcinoembryonic antigen (D, ×200)

Additionally, the patient had a 3-year history of erythematous skin rash of the scrotum, which had progressed during the two months prior to admission. The patient was referred to Division of Dermatology to undergo a biopsy of the genital area. Punch biopsy of the genital skin lesion showed a tumor located in the dermis, which was composed of malignant cuboidal cells arranged in solid lobules and tubules (Figure 1A). Small cystic lumina were noted within the infiltrative nests (Figure 1B). The cells had hyperchromatic nuclei with small nucleoli and moderate pleomorphism. Immunohistochemically, the tumor cells were diffusely positive for epithelial membrane antigen (EMA) and carcinoembryonic antigen (CEA; figures 1C and 1D). The tumor cells were negative for HMB-45 and S-100 protein. The patient was diagnosed with eccrine carcinoma.

**Figure 2:**
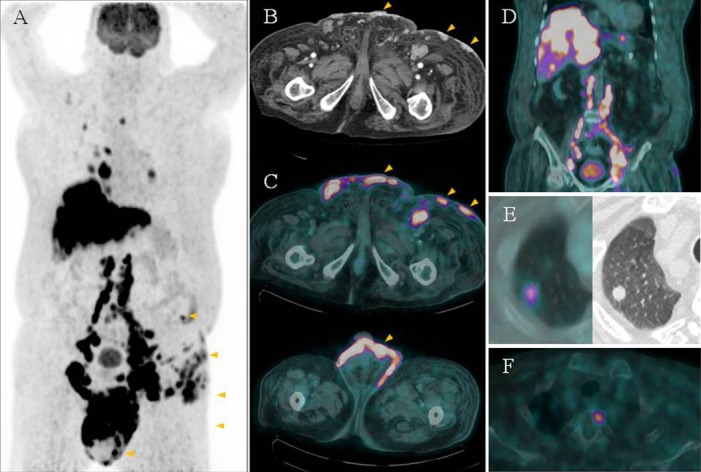
A 96-year-old man underwent F-18 FDG PET/CT to determine the initial clinical stage of his eccrine carcinoma. A maximal intensity projection image showed intense FDG uptake in multiple sites including the skin (arrowheads), nodes, liver, and lung. Axial enhanced CT (B) and fusion PET/CT (C) findings revealed varying degrees of FDG uptake over the groin, scrotum, penis, left pelvic wall, left hip, and left thigh (SUV_max_ 3.1-13.9), corresponding to enhancing skin thickening and nodules visualized on CT. D-F multiple FDG-avid distant metastatic lesions were also evident in the liver, lung, and bone.

The patient was referred for F-18 FDG PET/CT imaging to assess the extent of the disease. The procedure was performed 60 min after intravenous injection of 7.4 MBq/kg of F-18 FDG and 8 hours of fasting, using a Gemini TF PET/CT scanner (Philips Medical Systems, Cleveland, OH, USA). The maximal intensity projection image (Figure 2A) demonstrated extensive abnormal FDG-avid lesions in the body. Transaxial CT (Figure 2B) and PET/CT fusion images (Figure 2C) revealed enhanced nodular thickening and FDG uptake in all the clinically observed skin lesions (arrows). The maximum standardized uptake value (SUV_max_) of these lesions ranged from 3.1 to 13.9. Multiple FDG-avid metastatic lymph nodes were also observed in the mediastinum, retroperitoneal space, pelvis, and inguinal region (Figure 2D)Z. Extensive FDG-avid liver (Figure 2D), lung (Figure 2E), and bone (Figure 2F) metastatic lesions were also evident.

Eccrine carcinoma is traditionally managed with surgery, especially in the early stages. In the current case, wide surgical excision was ruled out because F-18 FDG PET/CT imaging successfully revealed an advanced disease stage. Chemotherapy and radiation therapy were considered. After receiving one week of palliative radiation therapy, the patient suffered from anemia, hypoproteinemia, and liver failure, which was possibly caused by the systemic metastases. Eventually, he expired from complications of radiation therapy.

## Discussion

Sweat gland carcinomas represent a rare group of tumors with the potential for destructive local tissue infiltration and both regional and distant metastases. The diagnosis and management of these neoplasms are both complex and cumbersome mainly due to lack of reports in the literature (5-8). Sweat gland carcinomas occur primarily in adult patients, with a peak incidence in the fifth and sixth decades of life (6, 9, 10). The majority of cases occur in genital skin and the perineum (34.5%), followed by the trunk (26.4%), head and neck (18.3%), and lower extremities (13.9%) (6, 8, 9, 11).

Eccrine carcinoma is a subtype of sweat gland carcinoma. Eccrine carcinomas possess no distinctive clinical features, making diagnosis by gross appearance virtually impossible. They usually manifest as non-tender, subcutaneous nodules, primarily in the elderly. Individual malignant cells are rich in glycogen and stain with PAS and are diastase sensitive with prevalent nuclear changes and propensity for lymphatic invasion (10, 11). Sites of sweat gland carcinoma metastasis include the nodes, lungs, liver, and bone (6, 11, 12). Metastatic deposits from undiagnosed visceral and breast adenocarcinoma are virtually indistinguishable microscopically from sweat gland carcinoma and must be considered before diagnosis of metastatic sweat gland carcinoma is made.

The recommended treatment for all subtypes of sweat gland carcinoma is wide surgical excision and regional lymph node dissection in the presence of clinically positive nodes. Some authors advocate prophylactic regional lymph node dissection, especially in patients with recurrent lesions after wide excision or highly undifferentiated tumors. Sweat gland carcinomas are radio-resistant, and chemotherapy is infrequently employed (13). Prognostic factors for sweat gland carcinomas are difficult to identify, again owing to the small number of reported cases. The likely prognostic factors include size, histological type, lymph node involvement, and distant metastasis. A 10-year disease-free survival rate of 56% in the absence of lymph node metastasis is observed, which falls to 9% if nodes are involved (6, 12, 13).

F-18 FDG-PET/CT is established as a valuable noninvasive imaging tool for diagnosis and staging, as well as a prognostic indicator for oncological patients. However, little is known about the use of F-18 FDG PET/CT imaging in the evaluation of eccrine sweat gland carcinoma. Increased FDG uptake in skin can also be nonspecific. Furthermore, it can mimic cutaneous or subcutaneous malignancies (cutaneous lymphoma, melanoma, and metastases from other internal malignancies) and an inflammatory or infective disease (14-19). However, the current case indicated that F-18 FDG PET/CT can be useful in evaluating eccrine sweat gland carcinoma by providing information about the extent of disease, lymph node involvement, and distant metastasis, all of which are closely related to the staging, management, and prognosis of the disease.
